# Molecular Characterization and Efficacy Evaluation of Transgenic Maize Harboring *cry2Ab*-*vip3A*-*cp4epsps* for Insect Resistance and Herbicide Tolerance

**DOI:** 10.3390/plants12030612

**Published:** 2023-01-30

**Authors:** Fantao Liu, Yuan Liu, Junjie Zou, Lan Zhang, Hongyan Zheng, Yanzhong Luo, Xiaoping Wang, Lei Wang

**Affiliations:** 1 Key Laboratory of Molecular Cytogenetics and Genetic Breeding of Heilongjiang Province, Harbin Normal University, Harbin 150025, China; 2 CAAS/Key Laboratory of Agricultural Genomics (Beijing), Biotechnology Research Institute, Ministry of Agriculture, Beijing 100081, China; 3 National Nanfan Research Institute (Sanya), Sanya 572022, China

**Keywords:** maize, insect resistance, herbicide tolerance, insect bioassay, insecticidal toxicity, glyphosate

## Abstract

Insect infestation and weed interference have a seriously negative impact on the growth, yield, and grain quality of maize. In this study, transgenic maize plants harboring three exogenous genes, *cry2Ab*, *vip3A*, and *cp4epsps*, that were constructed into a single T-DNA were developed for protection against insects and weeds. The transgene integration sites on the chromosomes in two transgenic maize events, CVC-1 and CVC-2, were determined using whole genome sequencing and specific PCR detection. As revealed by laboratory insect bioassays, these two transgenic events exhibited strong insecticidal toxicity against three major species of Lepidoptera insects, including *Mythimna separata*, *Helicoverpa armigera*, and *Spodoptera frugiperda*, with mortality rates exceeding 96%, 100%, and 100%, respectively, after six days of infestation. In addition, CVC-1 exhibited a high tolerance to glyphosate under field conditions. The successful expressions of *cry2Ab*, *vip3A*, and *cp4epsps* in various tissues at different developmental stages of CVC-1 were validated at the transcriptional and translational levels using quantitative real-time reverse transcription PCR (qRT-PCR) and enzyme-linked immunosorbent assay (ELISA), respectively. These findings demonstrated that the transgenic maize CVC-1 developed using this triple gene construct has excellent insect resistance and herbicide tolerance, which may provide a valuable germplasm resource and data support for future maize breeding of insect and weed control.

## 1. Introduction

Maize (*Zea mays* L.) is a globally important crop that serves as a source of food, animal feed, and industrial raw materials. Throughout the entire growth period, maize production is severely threatened by a variety of insect pests, and the attacks by these destructive pests cause substantial yield losses and quality compromises [1]. The oriental armyworm *Mythimna separata* (Walker), the bollworm *Helicoverpa armigera* (Hübner), and the fall armyworm *Spodoptera frugiperda* (J. E. Smith) are three major species of Lepidoptera insects that seriously affect global maize production. As a polyphagous, long-distance migratory, and sporadic corn pest, *M. separata* is notorious for its high feeding levels and can devour all available leaves when it abruptly outbreaks [2,3,4]. *H. armigera* is a widely distributed omnivorous pest that can migrate over long distances, and mainly feeds on leaves, silks, ears, and grains, thereby wreaking havoc on pollination, inducing ear rot, and reducing grain quality [2,5,6]. *S. frugiperda*, one of the most devastating pests of corn in America, has invaded a few provinces in China since 2019; it is a highly migratory and polyphagous pest that can cause significant damage to maize leaves, tassels, and ears, posing a major challenge for pest control [2,7,8]. Further, weed invasion represents another important cause of the considerable yield losses in maize, as field weeds often compete with the cultivated crops for nutrients, water, and light, as well as providing food and shelter for pests and plant pathogens [9,10]. The availability of identified genes that can confer plant resistance to pests and herbicides renders genetic engineering feasible for imparting these valuable traits to crops, which can greatly alleviate and control damages by pests and weeds.

Delta-endotoxins (Cry and Cyt) and vegetative insecticidal proteins (Vip) produced by *Bacillus thuringiensis* (Bt) have toxicity against insects mainly belonging to Lepidoptera, Diptera, and Coleoptera [11]. Although numerous Bt toxin proteins have been identified to date, only a few of them have been utilized in commercial Bt crops, such as Cry1Ab, Cry1Ac, Cry1F, Cry2Ab, Cry3, and Vip3A [12,13]. In contrast to the broad-spectrum insecticidal properties of chemical insecticides, these Bt proteins exhibited relatively narrow and selective toxicity against only a certain range of pests [2]. Cry2Ab shares no high-affinity receptors with Cry1A proteins in the insect midgut and displays toxicity against *M. separata* and *H. armigera* [2,14,15,16,17,18,19]. Vip3A does not share homology with Cry proteins, and its binding sites and mode of action are likewise distinct from those of Cry proteins [20,21,22]. It has been shown that Vip3A possesses toxic bioactivity towards lepidopteran pests, such as *M. separata*, *S. frugiperda*, *H. armigera*, and *Agrotis ypsilon* (Rottemberg) [2,23,24,25]. Compared to single Bt crops, transgenic crops that produce more than one Bt protein have a broader insecticidal spectrum and higher efficacy, and are more effective in delaying the development of pest resistance [2,13,19,26]. Thus, it is evident that the combination of discrete Bt toxins with the different binding sites on the insect midgut and modes of action in plants have the potential to confer perdurable resistance against a wide variety of pests. To date, however, there has been scant research on transgenic maize expressing the combination of *cry2Ab* and *vip3A* by constructing them into a single T-DNA and evaluating its efficacy against target insects, such as *M. separata*, *H. armigera*, and *S. frugiperda.*

Glyphosate, [*N*-(phosphonomethyl)glycine], is a broad-spectrum and potent herbicide that is widely used to eliminate weeds in the world. Glyphosate inhibits the 5-enolpyruvylshikimate-3-phosphate synthase (EPSPS) of the shikimate pathway, leading to the plants death by interfering in the biosynthesis of aromatic amino acids or causing shortages of carbon for other essential pathways due to the increased carbon flow to the shikimate pathway [27,28]. The *cp4epsps* gene is derived from the *Agrobacterium* sp. strain CP4, the protein product of which has natural tolerance to glyphosate [29]. Therefore, the expression of *cp4epsps* can confer glyphosate tolerance to transgenic plants, which represents a viable approach for the development of herbicide-tolerant transgenic crops.

In this study, three exogenous genes *cry2Ab*, *vip3A*, and *cp4epsps* were stacked to generate transgenic maize with robust insect resistance and herbicide tolerance. High-throughput genome sequencing and bioinformatics methods were used to determine the insertion sites of the transgenes in transgenic maize plants. The molecular characteristics were then analyzed using genomic PCR, real-time quantitative reverse transcription PCR (qRT-PCR), and enzyme-linked immunosorbent assay (ELISA) to determine the integration of the exogenous genes into the maize genome and the spatiotemporal profiles of their transcripts and proteins in various tissues during plant development. The insecticidal effects of the transgenic maize plants against *M. separata*, *H. armigera*, and *S. frugiperda*, as well as their tolerance to glyphosate, were evaluated using laboratory insect bioassays and field trials. The present study aimed to develop a triple-gene construct harboring *cry2Ab*, *vip3A*, and *cp4epsps* cassettes for maize transformation, as well as to evaluate the insect resistance and herbicide tolerance of the resulting transgenic maize.

## 2. Results

### 2.1. Development of Transgenic Plants Expressing cry2Ab, vip3A and cp4epsps

**Figure 1:**
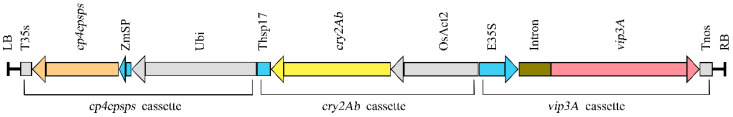
A schematic diagram of the T-DNA region harboring *cry2Ab*, *vip3A* and *cp4epsps* expression cassettes.LB: left border of T-DNA; T35s: CaMV35S terminator; *cp4epsps*: *cp4epsps* gene; ZmSP: maize EPSPS signal peptide; Ubi: maize ubiquitin promoter; Thsp17: heat shock protein Hsp17 terminator; *cry2Ab*: Bt insecticidal *cry2Ab* gene; OsAct2: rice Actin2 promoter; E35S: enhanced CaMV35S promoter; Intron: first intron of the rice *Actin* gene; *vip3A*: Bt *vip3A* gene; Tnos: nos terminator; RB: right border of T-DNA.

An expression cassette of the glyphosate-tolerant gene *cp4epsps* together with two functional cassettes of the insect-resistant genes *cry2Ab* and *vip3A* were constructed into a T-DNA under the transcriptional control of the maize Ubiquitin promoter (Ubi), the rice promoter Actin2 (OsAct2), and the enhanced CaMV35S promoter (E35S), respectively (Figure 1).

The T-DNA containing the three gene expression cassettes was then introduced into the recipient maize plants by *Agrobacterium*-mediated transformation. A total of 56 independent transgenic maize lines were obtained, among which twelve independent lines were found to express all three transgenes simultaneously with qualitative detection of protein expression using Cry2A, Vip3A, and CP4EPSPS immunoassay strips.

### 2.2. Identification of Integration Sites of Exogenous T-DNA in the Maize Genome

**Figure 2:**
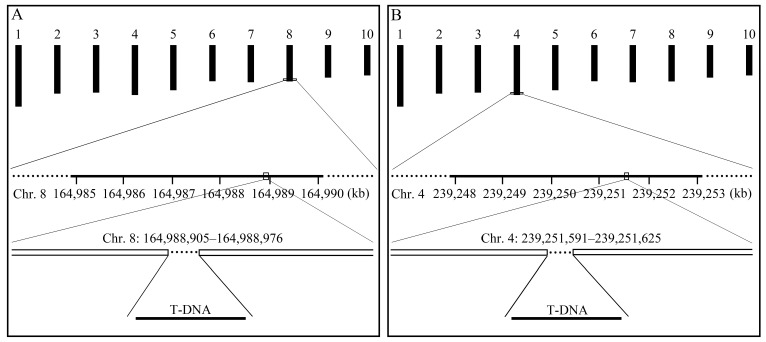
Schematic diagrams of the insertional T-DNA integrations in the CVC-1 (**A**) and CVC-2 (**B**) genomes. The upper part of the diagram displays ten chromosomes of maize. The numbers under the line of Chr.8 (**A**) and Chr.4 (**B**) indicate the physical positions on these chromosomes.

The whole genomes of the selected twelve transgenic lines were sequenced. More than 39.8 Gb of sequence data were generated using the Illumina platform for each of the twelve transgenic maize lines. The Phred quality score (Q) ≥ 30 of the sequencing data was greater than 92% for each line, demonstrating high data quality. The junction sequences with one end derived from the transformation plasmid sequence containing T-DNA and the other from the maize genome were selected for subsequent characterization of the insertion positions of the exogenous T-DNA in the maize genome [30,31]. Two independent transgenic lines with insertion sites in intergenic regions were designated as CVC-1 and CVC-2. In CVC-1, the exogenous T-DNA insertion site was located at positions 164,988,905–164,988,976 on chromosome 8. The integration caused several indels, including a 72-bp deletion of chromosomal DNA, followed by a 2-bp nucleotide insertion (Figure 2A). The T-DNA in CVC-2 was integrated into positions 239,251,591–239,251,625 on chromosome 4, resulting in a 35-bp deletion of chromosomal DNA (Figure 2B).

### 2.3. Analysis of Flanking Sequences and Specific PCR Detection

**Figure 3:**
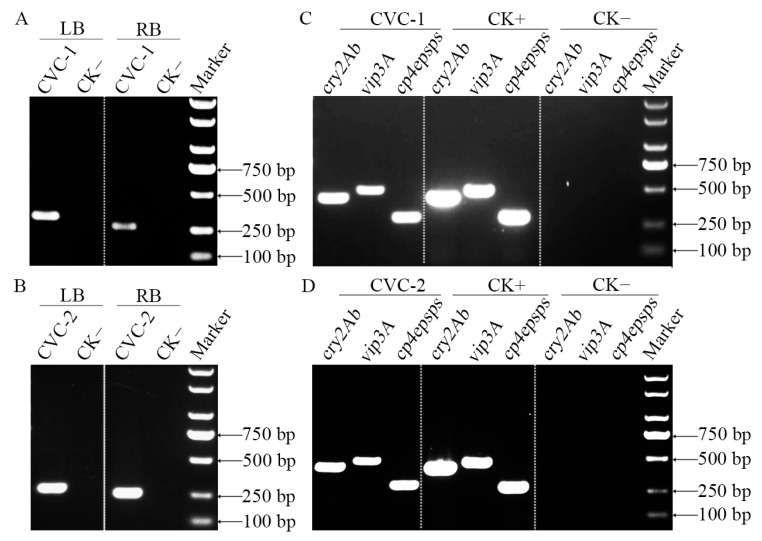
PCR specific identification of foreign T-DNA and target genes. The specific PCR identification of the flanking sequences of the transgenic maize CVC-1 (**A**) and CVC-2 (**B**); The PCR detection of *cry2Ab*, *vip3A*, and *cp4epsps* target genes in CVC-1 (**C**) and CVC-2 (**D**). LB: LB terminal flanking sequence; RB: RB terminal flanking sequence; CVC-1 and CVC-2: Transgenic maize events; CK+: Positive control of pL7-2 plasmid; CK−: Negative control of non-transgenic maize Zheng 58.

In order to confirm the precise site of T-DNA integration, the genomic DNA derived from CVC-1 was used as the PCR template, and two pairs of primers, T35LBfw2/CVC1LB-1 and NOsRBfw2/CVC1RB-1, were designed based on the DNA sequences at the junction of the left border (LB) terminal flanking sequence and T-DNA, and the junction of the right border (RB) terminal flanking sequence and T-DNA, respectively. Likewise, using CVC-2 genomic DNA as the template, the primer pairs T35LBfw1 + CVC2LB-1 and NOsRBfw2 + CVC2RB-1 were designed for the amplification of the LB and RB terminal flanking sequences, respectively. As shown in Figure 3A,B, PCR products of a single band with the expected size were obtained from both amplified LB and RB flanking sequences of CVC-1 and CVC-2. These flanking regions in CVC-1 and CVC-2 were subsequently sequenced and compared to the maize genome sequence and the inserted T-DNA sequence, validating the insertion position of the introduced T-DNAs in the transgenic maize genomes.

Transgene detection with PCR was performed on the transgenic CVC-1 and CVC-2 plants. The results showed that specific bands for the *cry2Ab*, *vip3A*, and *cp4epsps* genes were obtained, and the sizes of the resulting amplified DNA fragments were consistent with expectations (Figure 3C,D). These results demonstrate that the exogenous T-DNAs harboring the *cry2Ab*, *vip3A*, and *cp4epsps* genes were successfully integrated into the maize genome.

### 2.4. Insect Bioassays of Transgenic Maize against Three Lepidopteran Pests

To determine the insecticidal activity of Cry2Ab and Vip3A in the transgenic maize, *M. separata*, *H. armigera*, and *S. frugiperda* were selected as target pests for the laboratory insect bioassays. The results showed that the mortality rate of the neonate larvae of *M. separata* feeding on CVC-1 and CVC-2 leaves for 6 days (d) was greater than 96%, which was significantly higher than that of the control plant Zheng 58 (10.0%). After feeding for 4 d, the neonate larvae of *H. armigera* transferred on the CVC-1 leaves were all dead, whereas just a few of *H. armigera* transferred on the CVC-2 leaves were still alive after 4 d, but all were dead by 6 d. In contrast, the larval mortality rate of *H. armigera* that fed on the non-transgenic Zheng 58 maize leaves for 6 d was 52.1%. The neonate larvae of *S. frugiperda* that fed on the transgenic CVC-1 and CVC-2 leaves exhibited a mortality rate of 100% after 6 d infestation in contrast to 7.3% in the control plants (Table 1). The insect bioassays demonstrated that both transgenic maize lines, CVC-1 and CVC-2, exerted considerable insecticidal effects on *M. separata*, *H. armigera*, and *S. frugiperda*. By virtue of the mortality rates of the three pests and the extent of the damage to the transgenic maize leaves, the transgenic maize CVC-1 was chosen for further field herbicide tolerance evaluation and expression analyses.

### 2.5. Glyphosate Tolerance in Transgenic CVC-1 Fields

**Figure 4:**
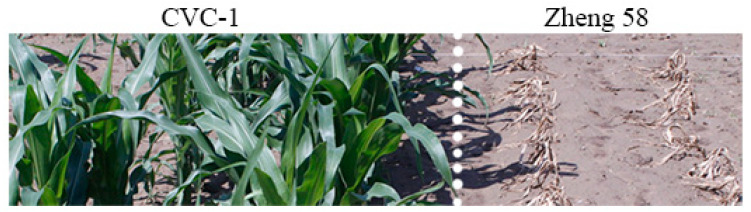
Glyphosate tolerance of the transgenic maize CVC-1 in the field. Non-transgenic maize Zheng 58 at the same growing stage was used as a control. The pictures were taken two weeks after glyphosate application.

To evaluate glyphosate tolerance in the transgenic maize expressing CP4EPSPS protein, the CVC-1 and non-transgenic control plants were sprayed with a glyphosate solution (10 mL/L) at a rate of 45 mL/m^2^, which was 2-fold of the recommended dose of maize field application. In sharp contrast to the control plants that were completely dead two weeks after glyphosate spraying, the CVC-1 plants exhibited an effective ability to cope with the influence of glyphosate and survive (Figure 4). It is therefore evident that CVC-1 was highly tolerant to glyphosate, and application of twice the recommended dose of glyphosate did not exert lethal effects on CVC-1.

### 2.6. Transcriptional Expression of Exogenous Target Genes in CVC-1

**Figure 5:**
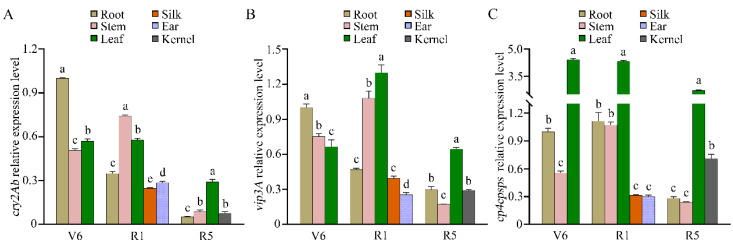
Relative expression levels of *cry2Ab* (**A**), *vip3A* (**B**), and *cp4epsps* (**C**) in the transgenic maize CVC-1. The data in all the samples are expressed as means ± standard deviation (*n* = 3). Different lowercase letters indicate significant differences among the plant tissues at the same developmental stages with Duncan’s multiple range test (one-way ANOVA; *p* < 0.05). V6: the six-leaf stage, R1: the silking stage, R5: the dent stage.

To comprehensively assess the expression patterns of *cry2Ab, vip3A*, and *cp4epsps* genes in CVC-1, various tissues at the V6 stage (six-leaf stage), R1 stage (silking stage) and R5 stage (dent stage) were chosen for qRT-PCR analysis (Figure 5). Results showed that the expression levels of *cry2Ab* were high in all tissues at the V6 and R1 stages, but relatively low in the roots, stems, and kernels at the R5 stage. The expression of *vip3A* in the leaves of the R1 stages was higher than those in the leaves of the V6 and R5 stages. Additionally, the accumulation of *vip3A* transcripts in the silks and the ears was lower than that in the stems and leaves at the R1 stage and was the lowest in the stems at the R5 stage. The expression levels of *cp4epsps* at each developmental stage were much higher in the leaves than in other tissues. These findings indicate that *cry2Ab, vip3A*, and *cp4epsps* can be transcribed in distinct tissues of CVC-1 at different developmental stages in a spatiotemporal manner.

### 2.7. Protein Expression of Exogenous Target Genes in CVC-1

**Figure 6:**
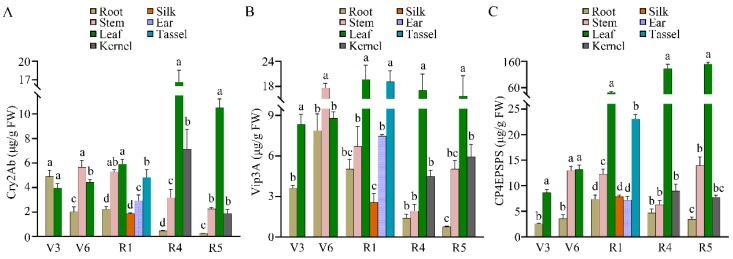
Protein expression levels of Cry2Ab (**A**), Vip3A (**B**), and CP4EPSPS (**C**) in the transgenic maize CVC-1. The data in all samples are presented as means ± standard deviation (*n* = 3). Different lowercase letters indicate significant differences among the plant tissues at the same developmental stages with Duncan’s multiple range test (one-way ANOVA; *p* < 0.05). FW, fresh weight of tissue. V3: the three-leaf stage, V6: the six-leaf stage, R1: the silking stage, R4: the dough stage, R5: the dent stage.

The expressed concentrations of Cry2Ab, Vip3A, and CP4EPSPS proteins in various tissues of CVC-1 at the V3 (three-leaf), V6, R1, R4 (dough), and R5 stages were determined with ELISA (Figure 6). There were high protein levels of Cry2Ab in the leaves and kernels at the R4 stage and the leaves at the R5 stage. Among them, Cry2Ab in the leaves at the R4 stage was the most abundant (16.6 ± 1.9 μg/g fresh weight (FW)), which was 2.3 times that in the kernels. The Vip3A content was relatively high in the stems at the V6 stage, tassels at the R1 stage, and leaves at the R4 and R5 stages; the highest concentration was found in the R1 leaves (19.6 ± 3.4 μg/g FW) in contrast to the lowest concentration in the roots at the R5 stage. The presence of CP4EPSPS was generally high in the leaves at the R1, R4, and R5 stages with the highest level being 149.7 ± 6.4 μg/g FW, which was 4.8, 15.3 and 17.3 times that in the V3 leaves, respectively. These results demonstrate that all three target genes in CVC-1 could be effectively translated into proteins in a spatially and temporally regulated manner.

## 3. Discussion

The determination and identification of the integration sites and the associated flanking sequences of T-DNA harboring the designated transgenes are the key to the event-specific detection in transgenic crops, which provides vital data required for the biosafety evaluation. With the development of high-throughput sequencing technology, whole genome sequences for transgenic crops can be quickly obtained at a relatively low cost [30,32]. In this study, using whole genome sequencing and bioinformatic methods, we demonstrated the integration of T-DNA harboring three transgene cassettes in the intergenic regions of maize chromosomes in two independent transgenic events, CVC-1 and CVC-2, which were further validated with PCR amplification and sequencing. Thus, our findings confirmed that combining whole genome sequencing with bioinformatics analysis is a simple and effective approach for identifying the integration sites for transgenic analysis [30,31,32]. Previous studies have demonstrated that different Bt transgenic events with distinct insertion sites exhibit a conspicuous divergence of efficacy in controlling target pests, which may be attributed to the varied expression levels of Bt genes [33,34]. Consistent with these findings, the two transgenic events, CVC-1 and CVC-2, exhibited variations in insect mortality for *M. separata, H. armigera*, *and S. frugiperda* in the insect bioassays in the present study. Furthermore, the successful expression of Bt transcripts and proteins in various plant tissues at different developmental stages is required for high and constant efficacy against insects. The normal expression of *cry2Ab* and *vip3A* in the tissues of the different developmental stages renders CVC-1 with the ability to prevent and control the damage of *M. separata*, *H. armigera*, and *S. frugiperda* to maize leaves, tassels, ears, and grains. Additionally, as an important agricultural pest, *A. ypsilon* can cause significant damage to corn stems and growth points and is difficult to control with chemical insecticides [2,35]. *A. ypsilon* has been reported to be more susceptible to Cry2Ab and Vip3A than Cry1 proteins [2,23]. Hence, the resistance of transgenic maize CVC-1 to *A. ypsilon* remains to be further investigated.

With more widespread adoption of Bt transgenic crops, particularly those containing a single Cry toxin, the long-term and extensive application of relatively high-dose insecticidal proteins also promotes the field-evolved insect resistance to Bt toxins under high selective pressure, which poses a serious threat to the durable effectiveness of Bt proteins [13,36,37,38]. The high dose refuge strategy has been used as a feasible and effective approach for insect resistance management in field transgenic crops [39,40]. In order to sustain the effectiveness of Bt crops against pests, the pyramiding or stacking approach that produce two or more Bt toxins is designed to delay and counter insect resistance, in conjunction with the use of a refuge [13,22,41,42]. The high enough concentration of each toxin in pyramided Bt crops to kill all or nearly all susceptible insects and no cross-resistance between them are especially important factors for slowing the evolution of insect resistance [13,19,22,42]. Cry2Ab and Vip3A proteins were both expressed at relatively high levels in the aboveground tissues of the transgenic maize CVC-1 throughout the developmental stages in this study. Compared to Cry proteins, to which field insects have been extensively exposed, the level of exposure to Vip proteins is limited due to their more recent deployment in Bt crops, and the field-evolved practical resistance to Vips has not been documented [19,43]. Further, there is no positive cross-resistance between the Vip3A and Cry2Ab proteins [21,43]. Additionally, Vip3A combined with Cry proteins exhibited a synergistic control effect with enhanced insecticidal activity against target insects [21]. Therefore, the simultaneous introduction of two different insect-resistant genes, *cry2Ab* and *vip3A*, to transgenic maize CVC-1 and even further combination with other toxins by hybridization of CVC-1 and other Bt maize can not only expand the efficacy spectrum and increase effective protection against pests, but also help to delay the development of field pest resistance and improve the durability of efficacy against target insects.

Weed interference poses a major threat to maize production. Previous studies have shown that overexpression of a modified EPSPS encoding gene *AM79 aroA* in transgenic maize confers excellent tolerance to glyphosate [44]. In this study, the glyphosate-resistance gene *cp4epsps* and the Bt *cry2Ab* and *vip3A* genes that were constructed in a single T-DNA were introduced together into maize. The *cp4epsps* gene can not only be used as a selection marker gene for transformation, but also confers resistance to the herbicide glyphosate. As revealed by qRT-PCR and ELISA analyses, the *cp4epsps* gene was efficiently expressed in various tissues at different developmental stages in the transgenic maize, demonstrating that the molecular stacking of insect-resistance and herbicide-tolerance genes in maize will not affect the successful expression of these genes. The experiments involving the application of glyphosate to plants in the field demonstrated that the transgenic maize CVC-1 was able to tolerate twice the recommended dose of glyphosate. Consistent with our results, a transgenic maize event ZD12-6 developed using a single T-DNA construct with a Bt fusion gene *Cry1Ab*/*Cry2Aj* and a modified *EPSPS* gene *G10* showed tolerance to twice the recommended dose of glyphosate [45]. Thus, it is plausible that simultaneously expressing the combination of the glyphosate-resistance genes and Bt genes in maize generated by the transgenic approach is effective in controlling weed invasion and minimizing field management costs.

In conclusion, the transgenic maize CVC-1, which successfully integrates and expresses the target genes *cry2Ab, vip3A*, and *cp4epsps* by constructing them in a single T-DNA, exhibits excellent insect resistance and herbicide tolerance, and has the potential application prospect for improving insect and weed control.

## 4. Materials and Methods

### 4.1. Vector Construction and Maize Transformation

The full-length coding sequences of *cp4epsps*, *cry2Ab*, and *vip3A* genes were all optimized according to the codon bias of maize and artificially synthesized and each of them was used to form its own expression cassette. The *cp4epsps* gene fused with the sequence of a maize EPSPS signal peptide was recombined under the transcriptional control of the synthesized maize ubiquitin promoter and CaMV35S *poly A* as a terminator at the *Mlu*I and *Spe*I restriction sites of pCAMBIA3301 (CAMBIA, Canberra, Australia). The *cry2Ab* coding region was inserted at the *Bam*HI and *Sac*I restriction sites between the synthesized OsAct2 promoter and heat shock protein Hsp17 terminator. The *vip3A* gene was inserted downstream of the synthesized enhanced CaMV35S promoter and the first intron of the rice *actin* gene and Tnos as a terminator using the *Sma*I and *Eco*RI restriction sites. Ultimately, three expression cassettes for *cry2Ab*, *vip3A*, and *cp4epsps* were successfully combined into a single plasmid, henceforth referred to as pL7-2, in the binary vector pCAMBIA3301 with the kanamycin selection, the T-DNA region of which is schematically presented in Figure 1. The final triple-gene construct *cry2Ab*-*vip3A*-*cp4epsps* in pL7-2 was verified by restriction analysis and ultimately transformed into *Agrobacterium tumefaciens* for stable integration into the plant genome.

As previously described [46], pL7-2 was transformed into the recipient maize, B104, using an *Agrobacterium*-mediated approach. The independent transgenic lines/events thus obtained were then crossed and consecutively backcrossed to an elite inbred line, Zheng58, and the exogenous inserted T-DNA was introduced into Zheng58 to obtain the transgenic maize CVC. The transgenic maize CVC lines and the non-transgenic control maize Zheng 58 were planted in a greenhouse or field of the Biotechnology Research Institute, Chinese Academy of Agricultural Sciences, under standard experimental practice, for further evaluation.

### 4.2. ImmunoStrip Test

The transgenic expression at the protein level was investigated using the ImmunoStrip^®^ test (Agdia, Elkhart, IN, USA). The Agdia products STX 05801/0050, STX 83500/0050, and STX 74000/0050 were used to assay Cry2A, Vip3A, and CP4EPSPS, respectively. Approximately 0.2 g of leaf samples derived from 56 transgenic lines was collected and ground to a fine slurry using pestles after adding ~500 μL extraction buffer (provided with Agdia products). The ImmunoStrip^®^ strip was then individually placed into the slurry and allowed to develop chromogenic bands for ~5 min.

### 4.3. Whole Genome Sequencing and Identification of the Insertion Sites of Transgenic Maize

Genomic DNA libraries from 12 transgenic maize lines were constructed according to the manufacturer’s protocol and subjected to sequencing on the Illumina platform. High-throughput sequence reads in a length of 150-bp were generated. Clean reads of each sample were aligned and mapped to the sequence of exogenous T-DNA from the transformation plasmid pL7-2 and maize reference genome B73 RefGen_v4 (Zm-B73-REFERENCE-GRAMENE-4.0) after adapter and low-quality reads were removed from the raw data. As previously described [30,31], the insertion loci of the exogenous T-DNA in the maize genome and the native genomic flanking sequences surrounding the insertion sites were identified and analyzed using Integrative Genomics Viewer (IGV_2.7.0) [47,48].

PCR primers (Table S1) were designed based on the flanking sequences at both ends of the integration site and the LB or RB sequences of the T-DNA. The PCR products were then sequenced to verify the insertion position of each transgenic line. PCR was performed in 20 μL of reaction mixtures containing ~100 ng of genomic DNA, 10 μL MonAmp™ 2× HS Taq Mix (+Dye) (Monad Biotech, China), and 375 nM each of the primers at the following conditions: 94 °C for 5 min, followed by 35 cycles of 94 °C for 30 s, 52~60 °C for 30 s, 72 °C for 30–60 s/kb, and a final extension at 72 °C for 5 min.

### 4.4. PCR Detection of Foreign Target Genes

The genomic DNAs of the transgenic maize lines, CVC-1 and CVC-2, and control maize Zheng 58 were extracted using the Cetyl-Trimethyl Ammonium Bromide (CTAB) method as described by Allen et al. (2006) [49] with a few modifications. The plasmid DNA of the expression vector pL7-2 was extracted using the FastPure EndoFree Plasmid Maxi Kit (Vazyme, Nanjing, China) following the manufacturer’s instructions. Based on the DNA sequences of the *cry2Ab, vip3A*, and *cp4epsps* genes, specific primers were designed (Table S1), and PCR detection was performed for the transgenic maize CVC-1 and CVC-2 lines as described above. Plasmid DNA was used as a positive control, and the Zheng 58 genomic DNA was used as a negative control.

### 4.5. Laboratory Insect Bioassays

For *M. separata*, fresh leaves at the V6 stage of the transgenic maize CVC-1 and CVC-2 were rinsed several times and blot dried. The leaves thus prepared were then transferred into a small worm box containing 20 neonate larvae and incubated under controlled conditions with a 16 h light and 8 h dark cycle, a relative humidity of 70%~80%, and an ambient temperature of 27 ± 1 °C. For *H. armigera* and *S. frugiperda*, fresh leaves derived from maize plants at the V6 stage were prepared as previously described. In addition, after removing the main vein from the leaf samples, they were cut into approximately 1 cm square leaflets. The leaflets and one newly hatched neonate larva were placed into each well of a 24-well culture plate, which was then incubated at the aforementioned controlled conditions.

The number of surviving larvae was recorded at a 2-d interval. Fresh leaves were added in time according to the condition of larvae feeding on the leaves during the experiment. During the assay, non-transgenic maize Zheng 58 was used as the negative control. Four replications were carried out for each insect species.

### 4.6. Evaluation of Glyphosate Tolerance of Transgenic Maize CVC-1

The field trial for glyphosate tolerance of the transgenic maize CVC-1 followed a split block design (plots of twin rows spaced ~0.6 m apart) with three replications. Glyphosate isopropylamine salt (41%, Roundup, Monsanto, St. Louis, MO, USA) diluted at 1:100 (10 mL/L) was sprayed onto the CVC-1 and non-transgenic control Zheng 58 plants at the 4–5 leaf stage with the rate of 45 mL/m^2^. The glyphosate tolerance was evaluated at two weeks after treatment.

### 4.7. Detection of Transcript Expression Levels with qRT-PCR

With qRT-PCR, the expression levels of *cry2Ab, vip3A*, and *cp4epsps* in the roots, stems, and leaves at the V6 stage; roots, stems, leaves, silks, and ears at the R1 stage; and roots, stems, leaves, and kernels at the R5 stage were evaluated.

In brief, RNAs were extracted from the samples with the RNA Easy Fast Plant Tissue Kit (TIANGEN, Beijing, China) and RNAprep Pure Plant Plus Kit (TIANGEN) following the manufacturer’s instructions. The first-strand cDNAs were then synthesized using MonScript™ RTIII All-in-One Mix with dsDNase (Monad Biotech). Each qRT-PCR was performed in a 20-μL reaction containing 10 μL TB Green Premix Ex Taq (TaKaRa, Tokyo, Japan), 0.4 μL ROX Reference Dye II, 1 μL of cDNA, and 200 nM each of the forward and reverse primers (Table S1). The reactions were carried out in a 7500 Real-Time PCR System (Applied Biosystems, Foster, CA, USA) with the following conditions: 95 °C for 30 s; followed by 40 cycles of 95 °C for 5 s, and 60 °C for 1 min. *ZmUbi2* was used as the endogenous reference gene [50]. The relative expression levels were calculated using the 2^−∆∆*C*_T_^ method [51].

### 4.8. Measurement of Protein Concentration with ELISA

Approximately 100 mg of roots, stems, leaves, silks, ears, tassels, and kernels at the different developmental stages was used to quantitatively analyze protein expression using the QualiPlate ELISA kits for Cry2Ab (Envirologix, Portland, ME, USA), Vip3A (Youlong Biotech, Shanghai, China), and CP4EPSPS (Youlong Biotech) following the manufacturers’ instructions, and the OD450 light absorption values were measured with a microplate reader (Synergy H1, BioTek, USA). The protein concentrations of Cry2Ab, Vip3A, and CP4EPSPS in each sample that were shown in μg/g fresh weight (FW) were determined according to the acquired OD450 values of the standard samples and the resulting standard curves.

### 4.9. Statistical Analysis

Statistical analyses were performed using SPSS 25.0 software. The differences in insect mortality were statistically assessed using analysis of variance (ANOVA) and the least significant difference test (LSD). For comparisons of the differences of transcript levels and protein contents among the plant tissues at the same developmental stages, Duncan’s multiple range tests (ANOVA) were carried out.

## Figures and Tables

**Table 1:** Mortality rates of *Mythimna separata*, *Helicoverpa armigera*, and *Spodoptera frugiperda* larvae after feeding on transgenic and non-transgenic control maize leaves (%).

Pests	Feeding Time/d	Mortality Rate of Larvae/%		
		CVC-1	CVC-2	Zheng 58
*Mythimna separata*	2 d	90.0 ± 7.1 b	88.8 ± 13.1 b	2.5 ± 2.9 a
	4 d	96.3 ± 2.5 b	96.3 ± 4.8 b	6.3 ± 4.8 a
	6 d	98.8 ± 2.5 b	96.3 ± 4.8 b	10.0 ± 4.1 a
*Helicoverpa armigera*	2 d	24.0 ± 12.4 b	21.9 ± 8.6 b	3.1 ± 6.3 a
	4 d	100.0 ± 0.0 b	99.0 ± 2.1 b	29.2 ± 9.6 a
	6 d	100.0 ± 0.0 b	100.0 ± 0.0 b	52.1 ± 9.9 a
*Spodoptera frugiperda*	2 d	9.4 ± 5.2 a	21.9 ± 8.6 b	4.2 ± 5.9 a
	4 d	100.0 ± 0.0 b	100.0 ± 0.0 b	7.3 ± 7.1 a
	6 d	100.0 ± 0.0 b	100.0 ± 0.0 b	7.3 ± 7.1 a

Note: Data in the table are presented as mean ± standard deviation (*n* = 4). Different lowercase letters in the same row indicate significant differences (ANOVA with LSD test, *p* < 0.05).

## Data Availability

The data are available on reasonable request from the corresponding author.

## Supplementary Materials

The following supporting information can be downloaded at: https://www.mdpi.com/article/10.3390/plants12030612/s1, Table S1: Primers used in this study.
