# Supplementary material for: Molecular Characterization and Efficacy Evaluation of Transgenic Maize Harboring cry2Ab-vip3A-cp4epsps for Insect Resistance and Herbicide Tolerance
Source: Plants (Basel). 2023 Jan 30;12(3):612. doi: 10.3390/plants12030612 (PMC9919038; doi:10.3390/plants12030612)
Supplement: Supplementary file 1 [file plants-12-00612-s001.zip › plants-2052149-supplementary.pdf]

Supplementary Materials:

Table S1 Primers used in this study

Primer name	Primer sequence (5'-3')	Purpose
T35LBfw2	TAATGTGTGAGTAGTTCCCAG	PCR identification of LB terminal flanking sequences of CVC-1
CVC1LB-1	CGTACGAGTATTCGCTGTAG	
NOsRBfw2	AGAGTCCCGCAATTATACAT	PCR identification of RB terminal flanking sequences of CVC-1
CVC1RB-1	TTCTTCCCGACAGCCAACAA	
T35LBfw2	TAATGTGTGAGTAGTTCCCAG	Sequencing validation of LB terminal flanking sequences of CVC-1
CVC1LB-2	ACATCAGTCGAAACGGCATC	
NOsRBfw2	AGAGTCCCGCAATTATACAT	Sequencing validation of RB terminal flanking sequences of CVC-1
CVC1RB-2	TGCTGGTCACTCTCTTTGCA	
T35LBfw1	GCTCATGTGTTGAGCATATAA	PCR identification of LB terminal flanking sequences of CVC-2
CVC2LB-1	AGAGTAGGAGATGGAATGGAAT	
NOsRBfw2	AGAGTCCCGCAATTATACAT	PCR identification of RB terminal flanking sequences of CVC-2
CVC2RB-1	ACGGTTACGGCAGCTAACT	
T35LBfw2	TAATGTGTGAGTAGTTCCCAG	Sequencing validation of LB terminal flanking sequences of CVC-2
CVC2LB-2	ATGGCTCTTTCCGCTACCT	
NOsRBfw2	AGAGTCCCGCAATTATACAT	Sequencing validation of RB terminal flanking sequences of CVC-2
CVC2RB-2	CACGCTGTCTAGGACAGAAA	
CP4-F	CTCAATGGCGTGGATTGCGAT	PCR identification of *cp4epsps* gene
CP4-R	CAGCCTTCGTATCGGAGAGTT	
Cry2Ab-F	ACCGTCACCAACTGGCAGAC	PCR identification of c*ry2Ab* gene
Cry2Ab-R	GGAGTCGCCCTGGTTGCCG	
Vip3A-F	GACTACCAGACCATCAACAA	PCR identification of *vip3A* gene
Vip3A-R	TTGATGCTCACGTCGTAGAA	
qZmUbi2F	TGGTTGTGGCTTCGTTGGTT	Reference gene, qRT-PCR
qZmUbi2R	GCTGCAGAAGAGTTTTGGGTACA	
Cry2Ab-F	ACCGTCACCAACTGGCAGAC	*cry2Ab*, qRT-PCR
qCry2Ab-R	TTGTAGTGCAGTGGACGGCGG	
qVip3A-F	AGGCGTTCCTGACCCTGAC	*vip3A*, qRT-PCR
qVip3A-R	TCATCTTCGCGTCCTCGTCG	
qCP4-F	ACCCATCTCGATCACCGCAT	*cp4epsps*, qRT-PCR
CP4-R	CAGCCTTCGTATCGGAGAGTT	
